# An Exploratory Study on the Development of an Animal Model of Acute Pancreatitis Following Nicotine Exposure

**DOI:** 10.1186/1617-9625-1-3-213

**Published:** 2003-09-15

**Authors:** P Chowdhury

**Affiliations:** 1 Department of Physiology & Biophysics, University of Arkansas for Medical Sciences

## Abstract

Cigarette smoking is known to be a major risk factor for pancreatic cancer and pancreatitis is believed to be a predisposed condition for pancreatic cancer. As of this date, there is no established experimental animal model to conduct detailed studies on these two deadly diseases. Our aim is to establish a rodent model by which we can systematically study the pathogenesis of pancreatitis and pancreatic cancer.

Adult Male Sprague Dawley rats were exposed to graded doses of nicotine by various routes for periods of three to 16 weeks. Blood samples were measured for hormonal and metabolic parameters. The pancreas was evaluated for histopathological changes and its function was assessed in isolated pancreatic acini upon stimulation with cholecystokinin (CCK) or carbachol (Cch). The pancreatic tissue was evaluated further for oncogene expression.

Body weight, food and fluid intakes, plasma glucose and insulin levels were significantly reduced in animals with nicotine exposure when compared to control. However, CCK and gastrin levels in the blood were significantly elevated. Pancreatic function was decreased significantly with no alteration in CCK receptor binding. Pancreatic histology revealed vacuolation, swelling, cellular pyknosis and karyorrhexis. Mutant oncogene, H-ras, was overexpressed in nicotine-treated pancreatic tissue.

The results suggest that alterations in metabolic, hormonal and pathologic parameters following nicotine-treatment appear consistent with diagnostic criteria of human pancreatitis. It is proposed that rats could be considered as a potential animal model to study the pathogenesis of pancreatitis.

## Introduction

Use of tobacco products, cigarettes and smokeless tobacco continues to be a major health problem in the United States and the world. Socio-economic and genetic factors may contribute to this problem [1,3,4]. It has been reported that an estimated 3 million deaths occur worldwide due to tobacco use [2]. Among the various components present in the tobacco products, nicotine and the dependency on it, appears to be the primary reason for the compulsive smoking behavior by smokers [5]. Therefore it may be surmised that nicotine is, at least partly responsible or may contribute as a major risk factor for pathogenesis of varieties of disorders in smoking population. The pathophysiological effects of smoking and nicotine on the pancreas have recently been reviewed [6].

Our current investigation focuses primarily on the exploration of the development of an animal model to study the development of two major pancreatic diseases, pancreatitis and pancreatic cancer, in response to nicotine exposure.

### Animals

Rats were used as the experimental model. The Institutional Animal Care and Use Committee at the University of Arkansas for Medical Sciences approved the protocols for the study. The animal study protocol followed the strict guidelines published by the American Physiological Society under Animal Welfare Act. All animals were procured two weeks prior to study, acclimatized with environmental conditions under 12/12 light dark cycle before being exposed to nicotine.

### Exposure conditions

Animals were weighed prior to being subjected to nicotine exposure. The animals were exposed to graded doses of nicotine for various lengths of time by three routes of administration (aerosol, intragastric and ad-lib drinking water). For example, while exposure by drinking water ad-libitum continued for 12 to 16 weeks, exposure by aerosol continued for 3 weeks with 1 hour daily exposure in the aerosol chamber. In contrast, nicotine given via intragastric administration by gavage feeding continued for 120 days with one administration per day. In all groups, consumption of daily food and fluid intake were measured. In all studies indicated, a paired identical control group, which was not exposed to nicotine, was taken for comparison. For each study, 6–8 animals per group were used.

At term, all animals were anesthetized with a mixture of ketamine hydrochloride and acyl-promazine mixture [(10:1 v/v): 0.1 ml per 100 g body weight)] and sacrificed by exsanguinations. Blood was collected in tubes containing heparin (10 ul, 10,000 units) and trasylol (10 ul, 100 KIE Units/ml). The blood was kept in ice until centrifuged at 3000 rpm. Plasma was separated and stored at -20°C for future analysis.

An abdominal incision was made to expose the pancreas. The pancreas was removed carefully, freed of fat and lymphatic vessels. A segment of the pancreas was fixed in 10% formalin in phosphate buffer, pH 7.4 for histopathological evaluation using the criteria published earlier by others [7-11]. The remaining pancreas was used for the isolation of acinar cells by the methods published previously by others and by us [12,13]. The isolated acinar cells were purified by differential centrifugation. Integrity of the pancreas (pancreatic function) was assessed by determining the ability of acinar cells to release amylase in response to graded doses of cholecystokinin.

### Assays

Plasma samples were assayed for gastrin, cholecystokinin and insulin by radioimmunoassays developed either in our laboratory or using the commercial kit [14,15]. Glucose levels were measured in the plasma by O-toluidine method [16]. Amylase release from the acinar cells was measured by employing procion yellow starch as substrate [17]. Protein concentration was determined by the method of Bradford [18]. CCK receptor assays were conducted in isolated acinar cell membranes using ^125^I-BH-CCK-8 employing the methods described by Steigerwalt and Williams [19]. CCK receptor affinity (Kd) and CCK receptor capacity (Bmax) were calculated from a Scatchard Plot developed by radio-ligand binding assay [20,21].

Oncogene expression and mutation was determined in fixed pancreatic tissues. DNA was isolated by enzyme digestion, amplified by polymerase chain reaction. Following electrophoresis and transfer to nylon filters, the filters were probed with ^32^P-cDNA-H-ras (0.8 kb) and K-ras (0.39 kb). For ras mutation experiments amplified PCR fragments obtained from codons 12, 13 and 61 were applied to prefixed nylon filters following denaturation at 95°C for 3 min. The filters were crosslinked using UV light, prehybridized and hybridized with specific radiolabeled oligonucleotides for each mutations at codons 12, 13 and 61. The filters were washed and analyzed on a betagen betascope autoradiography [22].

### Calculations

Data from different studies were summarized in the Results section. Comparison of the dataset between the groups was analyzed by student's t test or analysis of variance (ANOVA). A p value of less than 0.05 was considered statistically significant.

## Summary of Results

Body weights of the animals exposed to nicotine were significantly lower than the control animals at the time of sacrifice. Changes in metabolic parameters measured as an index of plasma glucose and insulin levels were significantly reduced in nicotine-treated groups than the control. However, the hormonal parameters (gastrin and CCK) were significantly elevated in nicotine-treated group as compared to control group.

Histopathological evaluation of the formalin fixed pancreatic tissues of the control animals indicated no gross ultrastructural changes. The pancreatic tissues from the nicotine exposed animals, however, revealed cytoplasmic swelling and vacuolation, pyknotic nuclei and karyorrhexis (fragmentation of nuclei). These pathologies remained strictly localized in the exocrine tissues of the pancreas.

Acinar cell bioassay experiments conducted with cells isolated from the control group in the presence of graded doses of CCK showed maximal stimulation of amylase release at a dose of 10^-10^M. The dose response curve shifted down to the right when repeated on acinar cells isolated from nicotine-treated animals. The maximal stimulated response by acinar cells isolated from nicotine-treated group was decreased significantly by as much as 50% from the control. There was no difference in total cellular amylase content (U amylase/mg of protein) between the two groups. The net retention of amylase following its release was higher in nicotine group as compared to control group.

There was no change in the acinar membrane binding parameters for CCK receptors between control and nicotine exposed groups. The changes in Kd and Bmax for CCK receptors between the two groups were not significantly different.

Oncogene studies indicated that both wild and mutant type oncoproteins were expressed in the pancreatic tissue. Frequency of mutations was higher in tissues from nicotine-treated rats when compared to that of control. DNA analysis of H-ras gene with amplifications of codons 12, 13 and 61 showed a specific mutation on codon 61 at glutamine of H-ras gene.

## Discussion

Reduction of body weights of animals exposed to nicotine is a complex event and perhaps regulated by increased energy utilization as reflected by the decreased plasma levels of glucose and insulin. It has been shown that smokers increase their energy utilization by increasing the basal metabolic rate [23,24]. Coupling with this observation is the decreased food intake that may have partly been influenced by high levels of endogenous CCK, which was shown to regulate the food intake [25].

Histopatholgical alterations noted in nicotine treated animals were consistent with the observations reported by other investigators in rats and other species [7-11] that indicates an earliest sign of the development of acute pancreatitis. Although cigarette smoking has been linked to chronic pancreatitis [6], the role of nicotine in this phenomenon has not been defined. The future directions of the study should implicate into the long-term pathological effects of nicotine on both exocrine and endocrine pancreas. The time course and dose dependent changes of pancreatic morphology from initiation to final development of pancreatitis need to be examined.

Functional changes of the pancreas in response to nicotine exposure were measured in isolated acinar cells with secretagogues that are known to stimulate the release of amylase or other enzymes [12,13]. A significant reduction of pancreatic function was noted with nicotine treatment that is consistent with the observations noted in patients with acute or chronic pancreatitis [8-11]. Similar loss of pancreatic function was reported in acute pancreatitis induced in other species by caerulein or choline deficient ethionine (CDE) diet [8-10]. Since no changes in CCK receptor affinity and capacity were noted in acinar membranes isolated from control and nicotine treated rats, it appears that loss of pancreatic function may have been associated with post-receptor mediated process.

Gene and point mutation studies on the pancreatic tissues indicated that nicotine exposure induced the activation and mutation of Ha-ras oncogene. Ras mutations have been implicated in many human cancers particularly in pancreatic cancers [26,27]. Activated K-ras gene has been noted in approximately 90% of human pancreatic carcinomas [27-29]. In a recent study with 97 Brazilian patients with pancreatic ductal adenocarcinoma, pancreatic neuroendocrine tumors and chronic pancreatitis, a K-ras mutation at codon 12 was found notably present in malignancies but absent in chronic pancreatitis and pancreatic neuroendocrine tumors [30]. The authors suggest consideration of the possibility of the treatment strategy for this oncogene in the future.

The data from our animal studies show a correlation between pathological, functional and molecular changes of the exocrine pancreatic tissue with nicotine exposure. These changes appear to reflect to some extent the pathophysiological events in acute pancreatitis. Using the time and duration of nicotine exposure as guides, the early phase and progression of induced pancreatic pathophysiological events can be documented, controlled and appropriate intervention may be instituted. The rodents may therefore be considered as a potential model for acute pancreatitis development following nicotine exposure and thus should be further explored.

## Competing interests

The authors declare that they have no competing interests.
